# An application of the Mittag-Leffler-type Borel distribution and Gegenbauer polynomials on a certain subclass of bi-univalent functions

**DOI:** 10.1016/j.heliyon.2024.e31469

**Published:** 2024-05-20

**Authors:** Abdulmtalb Hussen

**Affiliations:** School of Engineering, Math, and Technology, Navajo Technical University, Lowerpoint Rd State Hwy 371, Crownpoint, 87313 NM, United States

**Keywords:** Borel distribution, Mittag-Leffler, Gegenbauer polynomials, Bi-univalent functions, Analytic functions, Fekete–Szegö problem

## Abstract

The Mittag-Leffler-type Borel distribution is widely recognized and utilized as a beneficial and pertinent model across numerous applications. This study presents a new subclass of normalized analytic bi-univalent functions that combines Gegenbauer polynomials and the Mittag-Leffler-type Borel distribution. Employing this subclass enables us to derive novel approximations for Taylor-Maclaurin coefficients, $\left|a_{2}\right|$ and $\left|a_{3}\right|$, as well as delve into the investigation of the Fekete-Szegö functional. Additionally, we explore a variety of new findings that arise through the specialization of parameters in our primary results.

## 1 Introduction

Discrete probability distributions are mathematical models used to analyze the probabilities of different outcomes in situations where the sample space is countable or discrete. These distributions are widely employed in various fields such as statistics, mathematics, and computer science to understand and predict the occurrence of specific events. One particular type of discrete probability distribution is the Borel distribution, named after the renowned mathematician Emile Borel. The Borel distribution is often utilized in reliability theory and lifetime analysis, providing a framework to model rare events or extreme values. By utilizing the Borel distribution, researchers and analysts can effectively study and analyze discrete phenomena in real-life scenarios, enabling them to make informed decisions and draw meaningful conclusions based on the underlying probabilistic behavior.

A discrete random variable *τ* follows a Borel distribution with parameter μ∈[0,1] if its probability mass function is expressed as:

$$P(\tau =r)=\frac{{\left(\mu r\right)}^{r-1}e^{-\mu r}}{r!},\;r=1,2,3,\ldots \;\;.$$

Orthogonal polynomials have gained significant attention in recent years due to their importance in a wide range of disciplines. These polynomials hold notable interest as they frequently emerge as solutions to ordinary differential equations, under specific conditions outlined by various models. Among the classical orthogonal polynomials—Hermite, Laguerre, and Jacobi—encounters in practical applications are most frequent. Within the class of Jacobi polynomials, we find the Gegenbauer polynomials, which include well-known subclasses such as Legendre and Chebyshev polynomials. Those interested in exploring the fundamental definitions and essential properties of classical orthogonal polynomials are recommended to consult references [1], [2], [3]. Additionally, recent research has established connections between classical orthogonal polynomials and geometric function theory, which can be explored further in references [4], [5], [6], [7], [8], [9], [10], [11].

Gegenbauer polynomials (GPs), symbolized as $C_{n}(\tau )$, are a set of orthogonal polynomials defined on the interval I=[−1,1], considering the weight function ${\left(1-\tau^{2}\right)}^{\zeta -\frac{1}{2}}$, where $\zeta >-\frac{1}{2}$. They are recursively defined as follows:

(1.1) $$C_{0}^{\left(\zeta \right)}(\tau )=1,C_{1}^{\left(\zeta \right)}(\tau )=2\zeta \tau ,C_{n}^{\left(\zeta \right)}(\tau )=\frac{1}{n}[2\tau \left(n+\zeta -1\right)C_{n-1}^{\left(\zeta \right)}(\tau )-\left(n+2\zeta -2\right)C_{n-2}^{\left(\zeta \right)}(\tau )],\;\;\;n\geq 2.$$

In the case where we consider two distinct GPs, namely $C_{n}^{\left(\zeta \right)}(\tau )$ and $C_{m}^{\left(\zeta \right)}(\tau )$, with n≠m, we observe the following relationship:

$$\int_{-1}^{1}C_{n}^{\left(\zeta \right)}(\tau )\;C_{m}^{\left(\zeta \right)}(\tau )\;{\left(1-\tau^{2}\right)}^{\zeta -\frac{1}{2}}\;d\tau =0,$$

and with the case that n=m, we have

$$\int_{-1}^{1}{\left(C_{n}^{\left(\zeta \right)}(\tau )\right)}^{2}\;\;{\left(1-\tau^{2}\right)}^{\zeta -\frac{1}{2}}\;d\tau =\frac{\sqrt{\pi }\;\;\Gamma (n+2\zeta -1)}{2^{1-2\zeta }\;n!\;\Gamma (\zeta )\;\Gamma (n+\zeta -\frac{1}{2})}.$$

The generating function for the GP's is denoted as $G_{\zeta }(\tau ,\varphi )$ and is expressed through the following expansion:

(1.2) $$G_{\zeta }(\tau ,\varphi )=\frac{1}{{\left(1-2\tau \varphi +\varphi^{2}\right)}^{\zeta }}=\sum_{n=0}^{\infty }C_{n}^{\left(\zeta \right)}(\tau )\varphi^{n},\;\zeta >0,$$

where *τ* belongs to some interval *I*, and *φ* lies within the open unit disk U={φ:φ∈Cand|φ|<1}. When *τ* is fixed within *I*, $G_{\zeta }(\tau ,\varphi )$ is analytically defined in U and can be expanded into a Taylor series as described in equation (1.2). It's crucial to highlight that when ζ=0, $G_{\zeta }(\tau ,\varphi )$ yields no values.

The generating function of the GP is defined as follows:

$$G_{{}_{0}}(\tau ,\varphi )=1-\log\left(1-2\tau \varphi +\varphi^{2}\right)=\sum_{n=0}^{\infty }C_{n}^{\left(0\right)}(\tau )\;\varphi^{n}.$$

A solution to the Gegenbauer differential equation, known as a Gegenbauer polynomial of degree *n*, can be expressed by the following equation:

(1.3) $$(1-\tau^{2})\;\frac{d^{2}y}{d\tau^{2}}-(2\zeta +1)\tau \;\frac{dy}{d\tau }+n(n+2\zeta )\;y=0.$$

By setting $\zeta =\frac{1}{2}$ and ζ=1 in the equation (1.3), it transforms into the Legendre and Chebyshev differential equations correspondingly.

## 2 Preliminaries

Let's start this section by considering the class A, which consists of all analytic functions *f* defined within U and having the normalization conditions f(0)=0 and $f^{\prime }(0)=1$. For every f∈A, there exists a Taylor-Maclaurin series expansion in the form:

(2.1) $$f(\varphi )=\varphi +\sum_{n=2}^{\infty }a_{n}\;\varphi^{n},\;\;(\varphi \in U).$$

Additionally, let S denote the class of functions f∈A that are univalent within the unit disk U. We define two functions, *f* and *g*, to be subordinated, denoted as f≺g, if there exists an analytic function h(φ) (known as a Schwarz function) defined in U such that f(φ)=g(h(φ)), with the condition h(0)=0 and |h(φ)|≤1. Specifically, if the function *g* is univalent within U, then f(φ)≺g(φ)⇔f(0)=g(0)andf(U)⊂g(U) (see [12]).

As referenced in [13], there exists a principle known as the Koebe one-quarter theorem, indicating that any function *f* in the class S ensures that the unit disk U, under the transformation by *f*, contains a disk centered at the origin with a radius of $\frac{1}{4}$, expressed as $U_{\frac{1}{4}}(0)\subseteq f(U)$. Consequently, it follows that each function *f* within S possesses an inverse $f^{-1}$ mapping from f(U) back to U, subject to certain conditions:

$$f^{-1}(f(\varphi ))=\varphi ,\;(\varphi \in U)$$

and

$$f(f^{-1}(\sigma ))=\sigma ,\;(\left|\sigma \right|<r_{0}(f);\;\;\;r_{0}(f)\geq \frac{1}{4})$$

where $f^{-1}$ has the form:

(2.2) $$f^{-1}(\sigma )=g(\sigma )=\sigma -a_{{}_{2}}\;\sigma^{2}+(2a_{{}_{2}}^{2}-a_{{}_{3}})\;\sigma^{3}-(5a_{{}_{2}}^{3}-5a_{{}_{2}}a_{{}_{3}}+a_{{}_{4}})\;\sigma^{4}+\cdots .$$

In the unit disk U, a function f∈A is considered bi-univalent if both *f* and its inverse $f^{-1}$ are univalent. This class is denoted as Δ, defined by equation (2.1). For instance, functions like $f_{1}(\varphi )=\frac{\varphi }{1-\varphi }$ and $f_{2}(\varphi )=-\log(1-\varphi )$, along with their respective inverses, are examples of bi-univalent functions. The function $\text{K}(\varphi )=\frac{\varphi }{{\left(1-\varphi \right)}^{2}}$ is not in Δ because it maps the unit disk U to $C﹨(-\infty ,-\frac{1}{4}]$, which does not contain U (see [14], [15], [16], [17]). Additionally, other common univalent functions such as $g(\varphi )=\frac{\varphi }{1-\varphi^{2}}$ and $h(\varphi )=\varphi -\frac{\varphi^{2}}{2}$ are also not members of Δ.

Elementary distributions, a subset of generalized functions, are closely studied in geometric function theory. This branch of mathematics investigates the geometric properties of complex functions within specific domains. Theoretical investigations in this field have explored elementary distributions like Poisson, Pascal, Logarithmic, and Binomial distributions, aiming to understand their connections to geometric functions and explore their properties. These studies enhance our understanding of the interaction between elementary distributions, geometric functions, and their applications in mathematical analysis.

In a recent study by Wanas and Khuttar [18], a power series was formulated where the coefficients represent the probabilities associated with the Borel distribution as follows:

$$\Psi (\mu ,\varphi )=\varphi +\sum_{n=2}^{\infty }\frac{{\left(\mu (n-1)\right)}^{n-2}e^{-\mu (n-1)}}{(n-1)!}\varphi^{n},\;(\varphi \in U,\;\;0<\mu \leq 1).$$

The series mentioned above converges over the entire complex plane, validated by a well-established ratio test. Thus, this investigation addresses the normalization that follows the Mittag-Leffler function, a topic extensively discussed in references [19], [20], [21], [22].

$$K_{\beta ,\epsilon }(\varphi )=\varphi +\sum_{n=2}^{\infty }\frac{\Gamma (\epsilon )}{\Gamma (\beta (n-1)+\epsilon )}\varphi^{n},\;\varphi \in U,$$

where β,ϵ∈C;ϵ≠0,−1,−2,⋯; and Re(β)>0,Re(ϵ)>0.

By employing the concept of convolution (or Hadamard product) denoted as (⁎) for two analytic functions, we obtain:

$$\Pi_{\beta ,\epsilon }^{\mu }(\varphi )=K_{\beta ,\epsilon }(\varphi )⁎\Psi (\mu ,\varphi )=\varphi +\sum_{n=2}^{\infty }\frac{\Gamma (\epsilon ){\left(\mu (n-1)\right)}^{n-2}e^{-\mu (n-1)}}{\Gamma (\beta (n-1)+\epsilon )(n-1)!}\varphi^{n},$$

where 0<μ≤1,β,ϵ∈C;ϵ≠0,−1,−2,⋯; and Re(β)>0,Re(ϵ)>0.

Recently, Alatawi et al. [23] introduced a linear operator denoted as $V_{\beta ,\epsilon }^{\mu }(\varphi ):A\to A$ with the following definition:

$$V_{\beta ,\epsilon }^{\mu }f(\varphi )=\Pi_{\beta ,\epsilon }^{\mu }(\varphi )⁎f(\varphi )=\varphi +\sum_{n=2}^{\infty }\frac{\Gamma (\epsilon ){\left(\mu (n-1)\right)}^{n-2}e^{-\mu (n-1)}}{\Gamma (\beta (n-1)+\epsilon )(n-1)!}a_{n}\varphi^{n},$$

where 0<μ≤1,β,ϵ∈C;ϵ≠0,−1,−2,⋯ and Re(β)>0,Re(ϵ)>0.

Many researchers have focused on analyzing analytic and bi-univalent functions, particularly in relation to orthogonal polynomials and coefficient estimates. Within various subclasses of the bi-univalent function class Δ, proposed upper bounds for coefficients such as $\left|a_{2}\right|$ and $\left|a_{3}\right|$ have been presented [24], [25], [26]. Additionally, the Fekete-Szegö inequality, introduced by Fekete and Szegö in 1933 [27], provides a bound for the coefficients of univalent functions. This coefficient functional, pivotal in geometric function theory, is central to the Fekete-Szegö problem, which involves maximizing this functional. Recent research has yielded Fekete-Szegö inequalities for diverse function classes [28], [29], [30], [31]. Employing various probability distributions, such as Borel, Poisson, and Pascal distributions, researchers have studied specific subclasses of bi-univalent functions (examples are detailed in [32], [33], [34]). This study investigates the attributes of bi-univalent functions in connection with Gegenbauer polynomials and Borel distribution.

## 3 Bounding coefficients and estimating the Fekete–Szegö functional for the class $N_{\Delta }^{\lambda }(\beta ,\epsilon ,\mu ,G_{\zeta }(\tau ,\varphi ))$

This section introduces our new subclass of bi-univalent functions, denoted as $N_{\Delta }^{\lambda }(\beta ,\epsilon ,\mu ,G_{\zeta }(\tau ,\varphi ))$. Before proceeding further, we first recall a useful lemma and then present the main result, which offers coefficient estimates for our defined class.

Definition 3.1 Let λ∈[0,1] and $\tau \in (\frac{1}{2},1]$. A function f∈Δ given by (2.1) is considered to be in the class $N_{\Delta }^{\lambda }(\beta ,\epsilon ,\mu ,G_{\zeta }(\tau ,\varphi ))$ with a nonzero real constant *ζ* if the following subordinations are satisfied:

(3.1) $$\lambda \left(1+\frac{\varphi \;{\left(V_{\beta ,\epsilon }^{\mu }f(\varphi )\right)}^{\prime \prime }}{{\left(V_{\beta ,\epsilon }^{\mu }f(\varphi )\right)}^{\prime }}\right)+(1-\lambda )\left(\frac{\varphi \;{\left(V_{\beta ,\epsilon }^{\mu }f(\varphi )\right)}^{\prime }}{V_{\beta ,\epsilon }^{\mu }f(\varphi )}\right)≺G_{{}_{\zeta }}(\tau ,\varphi ),$$

and

(3.2) $$\lambda \left(1+\frac{\sigma \;{\left(V_{\beta ,\epsilon }^{\mu }g(\sigma )\right)}^{\prime \prime }}{{\left(V_{\beta ,\epsilon }^{\mu }g(\sigma )\right)}^{\prime }}\right)+(1-\lambda )\left(\frac{\sigma \;{\left(V_{\beta ,\epsilon }^{\mu }g(\sigma )\right)}^{\prime }}{V_{\beta ,\epsilon }^{\mu }g(\sigma )}\right)≺G_{{}_{\zeta }}(\tau ,\sigma ),$$

where $G_{{}_{\zeta }}$ is defined by equation (1.2) and the function g(σ) is defined by equation (2.2).

Example 3.1 Let *f* be a bi-univalent function belonging to the class Δ. We say that *f* is in the class $N_{\Delta }^{0}(\beta ,\epsilon ,\mu ,G_{\zeta }(\tau ,\varphi ))$ if the subsequent subordination conditions are satisfied:

$$\frac{\varphi \;{\left(V_{\beta ,\epsilon }^{\mu }f(\varphi )\right)}^{\prime }}{V_{\beta ,\epsilon }^{\mu }f(\varphi )}≺G_{\zeta }(\tau ,\varphi )$$

and

$$\frac{\sigma \;{\left(V_{\beta ,\epsilon }^{\mu }g(\sigma )\right)}^{\prime }}{V_{\beta ,\epsilon }^{\mu }g(\sigma )}≺G_{\zeta }(\tau ,\sigma ),$$

where the function g(σ) is defined by equation (2.2).

Example 3.2 Let *f* be a bi-univalent function belonging to the class Δ. We say that *f* is in the class $N_{\Delta }^{1}(\beta ,\epsilon ,\mu ,G_{\zeta }(\tau ,\varphi ))$, if the subsequent subordination conditions are satisfied:

$$1+\frac{\varphi \;{\left(V_{\beta ,\epsilon }^{\mu }f(\varphi )\right)}^{\prime \prime }}{{\left(V_{\beta ,\epsilon }^{\mu }f(\varphi )\right)}^{\prime }}≺G_{\zeta }(\tau ,\varphi ),$$

and

$$1+\frac{\sigma \;{\left(V_{\beta ,\epsilon }^{\mu }g(\sigma )\right)}^{\prime \prime }}{{\left(V_{\beta ,\epsilon }^{\mu }g(\sigma )\right)}^{\prime }}≺G_{\zeta }(\tau ,\sigma ),$$

where the function g(σ) is defined by equation (2.2).

Lemma 3.1 *[13]* *Let* ω∈Ω *with* $\omega (\varphi )=\sum_{n=1}^{\infty }\omega_{n}\varphi^{n},\;\;\;\varphi \in U$ *. Then,*

$$\left|\omega_{1}\right|\leq 1,\;\left|\omega_{n}\right|\leq 1-{\left|\omega_{1}\right|}^{2}\;\;\;\;\;for\;\;\;\;\;n\in N﹨\{1\}.$$

Theorem 3.1 *Let* f∈Δ *of the form* (2.1) *be in the class* $N_{\Delta }^{\lambda }(\beta ,\epsilon ,\mu ,G_{\zeta }(\tau ,\varphi ))$ *. Then,*

$$\left|a_{2}\right|\leq \frac{2\tau |\zeta |e^{\mu }\Gamma (\beta +\epsilon )\sqrt{2\tau |\zeta |\Gamma (2\beta +\epsilon )}}{\sqrt{\begin{array}{c} \left|{\left(2\zeta \tau \right)}^{2}\Gamma (\epsilon )(2\mu (1+2\lambda ){\left(\Gamma (\beta +\epsilon )\right)}^{2}-(1+3\lambda )\Gamma (2\beta +\epsilon )\Gamma (\epsilon ))-(2\zeta (1+\zeta )\tau^{2}-\zeta ){\left(1+\lambda \right)}^{2}\Gamma (2\beta +\epsilon ){\left(\Gamma (\epsilon )\right)}^{2}\right| \end{array}}},$$

*and*

$$\left|a_{3}\right|\leq \frac{8\tau^{3}|\zeta {|}^{3}e^{2\mu }{\left(\Gamma (\beta +\epsilon )\right)}^{2}\Gamma (2\beta +\epsilon )}{\begin{array}{c} \left|{\left(2\zeta \tau \right)}^{2}\Gamma (\epsilon )(2\mu (1+2\lambda ){\left(\Gamma (\beta +\epsilon )\right)}^{2}-(1+3\lambda )\Gamma (2\beta +\epsilon )\Gamma (\epsilon ))-(2\zeta (1+\zeta )\tau^{2}-\zeta ){\left(1+\lambda \right)}^{2}\Gamma (2\beta +\epsilon ){\left(\Gamma (\epsilon )\right)}^{2}\right| \end{array}}+\frac{\tau |\zeta |\Gamma (2\beta +\epsilon )e^{2\mu }}{\mu (1+2\lambda )\Gamma (\epsilon )}.$$

Proof Let $f\in N_{\Delta }^{\lambda }(\beta ,\epsilon ,\mu ,G_{\zeta }(\tau ,\varphi ))$ for some 0≤λ≤1, and from (3.1) and (3.2) we obtain

(3.3) $$\lambda \left(1+\frac{\varphi \;{\left(V_{\beta ,\epsilon }^{\mu }f(\varphi )\right)}^{\prime \prime }}{{\left(V_{\beta ,\epsilon }^{\mu }f(\varphi )\right)}^{\prime }}\right)+(1-\lambda )\left(\frac{\varphi \;{\left(V_{\beta ,\epsilon }^{\mu }f(\varphi )\right)}^{\prime }}{V_{\beta ,\epsilon }^{\mu }f(\varphi )}\right)=G_{\zeta }(\tau ,u(\varphi )),$$

and

(3.4) $$\lambda \left(1+\frac{\sigma \;{\left(V_{\beta ,\epsilon }^{\mu }g(\sigma )\right)}^{\prime \prime }}{{\left(V_{\beta ,\epsilon }^{\mu }g(\sigma )\right)}^{\prime }}\right)+(1-\lambda )\left(\frac{\sigma \;{\left(V_{\beta ,\epsilon }^{\mu }g(\sigma )\right)}^{\prime }}{V_{\beta ,\epsilon }^{\mu }g(\sigma )}\right)=G_{\zeta }(\tau ,v(\sigma )),$$

where $g(\sigma )=f^{-1}(\sigma )$ and u,v∈Ω are given to be of the form

$$u(\varphi )=\sum_{n=1}^{\infty }c_{n}\;\varphi^{n}\;\;\;\;\;\;\;\;\;\;and\;\;\;\;\;\;\;\;\;\;v(\sigma )=\sum_{n=1}^{\infty }d_{n}\;\sigma^{n}.$$

From Lemma 3.1, we have

(3.5) $$\left|c_{n}\right|\leq 1\;\text{ and }\;\left|d_{n}\right|\leq 1,\;\;n\in N.$$

By employing the provided generating function, denoted as $G_{\zeta }(\tau ,\varphi )$ in equation (1.2), we can show that the expressions on the right-hand sides of equations (3.3) and (3.4) take the following form:

$$G_{\zeta }(\tau ,u(\varphi ))=1+C_{1}^{\zeta }(\tau )c_{1}\varphi +\left[C_{1}^{\zeta }(\tau )c_{2}+C_{2}^{\zeta }(\tau )c_{1}^{2}\right]\varphi^{2}+\cdots ,$$

and

$$G_{\zeta }(\tau ,v(\sigma ))=1+C_{1}^{\zeta }(\tau )d_{1}\sigma +\left[C_{1}^{\zeta }(\tau )d_{2}+C_{2}^{\zeta }(\tau )d_{1}^{2}\right]\sigma^{2}+\cdots .$$

Therefore, (3.3) and (3.4) become

(3.6) $$1+[(1+\lambda )\frac{\Gamma (\epsilon )e^{-\mu }}{\Gamma (\beta +\epsilon )}a_{2}]\varphi +[2(1+2\lambda )\frac{\Gamma (\epsilon )\mu e^{-2\mu }}{\Gamma (2\beta +\epsilon )}a_{3}-(1+3\lambda ){\left(\frac{\Gamma (\epsilon )e^{-\mu }}{\Gamma (\beta +\epsilon )}\right)}^{2}a_{2}^{2}]\varphi^{2}=1+C_{1}^{\zeta }(\tau )c_{1}\varphi +[C_{1}^{\zeta }(\tau )c_{2}+C_{2}^{\zeta }(\tau )c_{1}^{2}]\varphi^{2}+\cdots ,$$

and

(3.7) $$1-[(1+\lambda )\frac{\Gamma (\epsilon )e^{-\mu }}{\Gamma (\beta +\epsilon )}a_{2}]\sigma +[(4(1+2\lambda )\frac{\Gamma (\epsilon )\mu e^{-2\mu }}{\Gamma (2\beta +\epsilon )}-(1+3\lambda ){\left(\frac{\Gamma (\epsilon )e^{-\mu }}{\Gamma (\beta +\epsilon )}\right)}^{2})a_{2}^{2}-2(1+2\lambda )\frac{\Gamma (\epsilon )\mu e^{-2\mu }}{\Gamma (2\beta +\epsilon )}a_{3}]\sigma^{2}+\cdots =1+C_{1}^{\zeta }(\tau )d_{1}\sigma +[C_{1}^{\zeta }(\tau )d_{2}+C_{2}^{\zeta }(\tau )d_{1}^{2}]\sigma^{2}+\cdots .$$

By comparing the coefficients in equations (3.6) and (3.7), we observe that

(3.8) $$(1+\lambda )\frac{\Gamma (\epsilon )e^{-\mu }}{\Gamma (\beta +\epsilon )}a_{2}=C_{1}^{\zeta }(\tau )c_{1},$$

(3.9) $$2(1+2\lambda )\frac{\Gamma (\epsilon )\mu e^{-2\mu }}{\Gamma (2\beta +\epsilon )}a_{3}-(1+3\lambda ){\left(\frac{\Gamma (\epsilon )e^{-\mu }}{\Gamma (\beta +\epsilon )}\right)}^{2}a_{2}^{2}=C_{1}^{\zeta }(\tau )c_{2}+C_{2}^{\zeta }(\tau )c_{1}^{2},$$

(3.10) $$-(1+\lambda )\frac{\Gamma (\epsilon )e^{-\mu }}{\Gamma (\beta +\epsilon )}a_{2}=C_{1}^{\zeta }(\tau )d_{1},$$

and

(3.11) $$(4(1+2\lambda )\frac{\Gamma (\epsilon )\mu e^{-2\mu }}{\Gamma (2\beta +\epsilon )}-(1+3\lambda ){\left(\frac{\Gamma (\epsilon )e^{-\mu }}{\Gamma (\beta +\epsilon )}\right)}^{2})a_{2}^{2}-2(1+2\lambda )\frac{\Gamma (\epsilon )\mu e^{-2\mu }}{\Gamma (2\beta +\epsilon )}a_{3}=C_{1}^{\zeta }(\tau )d_{2}+C_{2}^{\zeta }(\tau )d_{1}^{2}.$$

From equations (3.8) and (3.10), we can derive the following two equations:

$$c_{1}=-d_{1}$$

and

(3.12) $$c_{1}^{2}+d_{1}^{2}=2{\left(\frac{\left(1+\lambda )\Gamma (\epsilon \right)}{e^{\mu }\Gamma (\beta +\epsilon )C_{1}^{\zeta }(\tau )}\right)}^{2}a_{2}^{2},$$

from equations (3.9), (3.11), and (3.12), we can deduce the following:

(3.13) $$a_{2}^{2}=\frac{{\left(C_{1}^{\zeta }(\tau )\right)}^{3}e^{2\mu }\Gamma (2\beta +\epsilon ){\left(\Gamma (\beta +\epsilon )\right)}^{2}(c_{2}+d_{2})}{\begin{array}{c} 2{\left(C_{1}^{\zeta }(\tau )\right)}^{2}\Gamma (\epsilon )(2\mu (1+2\lambda ){\left(\Gamma (\beta +\epsilon )\right)}^{2}-(1+3\lambda )\Gamma (2\beta +\epsilon )\Gamma (\epsilon ))-2C_{2}^{\zeta }(\tau ){\left(1+\lambda \right)}^{2}\Gamma (2\beta +\epsilon ){\left(\Gamma (\epsilon )\right)}^{2} \end{array}}.$$

By taking the absolute value of equation (3.13) and utilizing equation (3.5), we derive the following:

$${\left|a_{2}\right|}^{2}\leq \frac{{\left|C_{1}^{\zeta }(\tau )\right|}^{3}e^{2\mu }\Gamma (2\beta +\epsilon ){\left(\Gamma (\beta +\epsilon )\right)}^{2}}{\begin{array}{c} \left|{\left(C_{1}^{\zeta }(\tau )\right)}^{2}\Gamma (\epsilon )(2\mu (1+2\lambda ){\left(\Gamma (\beta +\epsilon )\right)}^{2}-(1+3\lambda )\Gamma (2\beta +\epsilon )\Gamma (\epsilon ))-C_{2}^{\zeta }(\tau ){\left(1+\lambda \right)}^{2}\Gamma (2\beta +\epsilon ){\left(\Gamma (\epsilon )\right)}^{2}\right| \end{array}},$$

and therefore

(3.14) $$\left|a_{2}\right|\leq \frac{e^{\mu }\Gamma (\beta +\epsilon )\left|C_{1}^{\zeta }(\tau )\right|\sqrt{\left|C_{1}^{\zeta }(\tau )\right|\Gamma (2\beta +\epsilon )}}{\sqrt{\begin{array}{c} \left|{\left(C_{1}^{\zeta }(\tau )\right)}^{2}\Gamma (\epsilon )(2\mu (1+2\lambda ){\left(\Gamma (\beta +\epsilon )\right)}^{2}-(1+3\lambda )\Gamma (2\beta +\epsilon )\Gamma (\epsilon ))-C_{2}^{\zeta }(\tau ){\left(1+\lambda \right)}^{2}\Gamma (2\beta +\epsilon ){\left(\Gamma (\epsilon )\right)}^{2}\right| \end{array}}}.$$

Substituting $C_{1}^{\zeta }(\tau )$ and $C_{2}^{\zeta }(\tau )$ as given in (1.1) into equation (3.14), we obtain the following result:

$$\left|a_{2}\right|\leq \frac{2\tau |\zeta |e^{\mu }\Gamma (\beta +\epsilon )\sqrt{2\tau |\zeta |\Gamma (2\beta +\epsilon )}}{\sqrt{\begin{array}{c} \left|{\left(2\zeta \tau \right)}^{2}\Gamma (\epsilon )(2\mu (1+2\lambda ){\left(\Gamma (\beta +\epsilon )\right)}^{2}-(1+3\lambda )\Gamma (2\beta +\epsilon )\Gamma (\epsilon ))-(2\zeta (1+\zeta )\tau^{2}-\zeta ){\left(1+\lambda \right)}^{2}\Gamma (2\beta +\epsilon ){\left(\Gamma (\epsilon )\right)}^{2}\right| \end{array}}}.$$

To estimate $\left|a_{3}\right|$, we start by subtracting equation (3.11) from equation (3.9)

(3.15) $$a_{3}=a_{2}^{2}+\frac{C_{1}^{\zeta }(\tau )\Gamma (2\beta +\epsilon )e^{2\mu }(c_{2}-d_{2})}{4\mu (1+2\lambda )\Gamma (\epsilon )},$$

as a result, we obtain the following expression:

$$\left|a_{3}\right|\leq {\left|a_{2}\right|}^{2}+\frac{\left|C_{1}^{\zeta }(\tau )\right|\Gamma (2\beta +\epsilon )e^{2\mu }\left|c_{2}-d_{2}\right|}{4\mu (1+2\lambda )\Gamma (\epsilon )}.$$

By utilizing the inequalities (3.5) and equation (1.1), we can derive the following:

$$\left|a_{3}\right|\leq \frac{8\tau^{3}|\zeta {|}^{3}e^{2\mu }{\left(\Gamma (\beta +\epsilon )\right)}^{2}\Gamma (2\beta +\epsilon )}{\begin{array}{c} \left|{\left(2\zeta \tau \right)}^{2}\Gamma (\epsilon )(2\mu (1+2\lambda ){\left(\Gamma (\beta +\epsilon )\right)}^{2}-(1+3\lambda )\Gamma (2\beta +\epsilon )\Gamma (\epsilon ))-(2\zeta (1+\zeta )\tau^{2}-\zeta ){\left(1+\lambda \right)}^{2}\Gamma (2\beta +\epsilon ){\left(\Gamma (\epsilon )\right)}^{2}\right| \end{array}}+\frac{\tau |\zeta |\Gamma (2\beta +\epsilon )e^{2\mu }}{\mu (1+2\lambda )\Gamma (\epsilon )}.$$

 □

By utilizing the values of $a_{2}^{2}$ and $a_{3}$, we can easily establish the following Fekete–Szegö inequality for functions $f\in N_{\Delta }^{\lambda }(\beta ,\epsilon ,\mu ,G_{\zeta }(\tau ,\varphi ))$.

Theorem 3.2 *Let*f∈Δ*given by the form*(2.1)*be in the class*$N_{\Delta }^{\lambda }(\beta ,\epsilon ,\mu ,G_{\zeta }(\tau ,\varphi ))$*. Then,*

$$\left|a_{3}-\eta a_{2}^{2}\right|\leq \left\{ \begin{array}{cc} \frac{\tau \left|\zeta \right|\Gamma (2\beta +\epsilon )e^{2\mu }}{\mu (1+2\lambda )\Gamma (\epsilon )} & \;\text{if}\;0\leq \left|h(\eta )\right|\leq \frac{\Gamma (2\beta +\epsilon )e^{2\mu }}{4\mu (1+2\lambda )\Gamma (\epsilon )}\\ 4\tau \left|\zeta \right|\left|h(\eta )\right| & \;\text{if}\;\left|h(\eta )\right|\geq \frac{\Gamma (2\beta +\epsilon )e^{2\mu }}{4\mu (1+2\lambda )\Gamma (\epsilon )}, \end{array}\right.$$

*where*

$$h(\eta )=\frac{(1-\eta ){\left(C_{1}^{\zeta }(\tau )\right)}^{2}e^{2\mu }\Gamma (2\beta +\epsilon ){\left(\Gamma (\beta +\epsilon )\right)}^{2}}{\begin{array}{c} 2{\left(C_{1}^{\zeta }(\tau )\right)}^{2}\Gamma (\epsilon )(2\mu (1+2\lambda ){\left(\Gamma (\beta +\epsilon )\right)}^{2}-(1+3\lambda )\Gamma (2\beta +\epsilon )\Gamma (\epsilon ))-2C_{2}^{\zeta }(\tau ){\left(1+\lambda \right)}^{2}\Gamma (2\beta +\epsilon ){\left(\Gamma (\epsilon )\right)}^{2} \end{array}}.$$

Proof From equations (3.13) and (3.15), we have

$$\begin{array}{cc} a_{3}-\eta a_{2}^{2} & =a_{2}^{2}+\frac{C_{1}^{\zeta }(\tau )\Gamma (2\beta +\epsilon )e^{2\mu }(c_{2}-d_{2})}{4\mu (1+2\lambda )\Gamma (\epsilon )}-\eta a_{2}^{2}\\ & =(1-\eta )a_{2}^{2}+\frac{C_{1}^{\zeta }(\tau )\Gamma (2\beta +\epsilon )e^{2\mu }(c_{2}-d_{2})}{4\mu (1+2\lambda )\Gamma (\epsilon )}\\ & =\frac{\left(1-\eta ){\left(C_{1}^{\zeta }(\tau )\right)}^{3}e^{2\mu }\Gamma (2\beta +\epsilon ){\left(\Gamma (\beta +\epsilon )\right)}^{2}(c_{2}+d_{2}\right)}{\begin{array}{c} 2{\left(C_{1}^{\zeta }(\tau )\right)}^{2}\Gamma (\epsilon )(2\mu (1+2\lambda ){\left(\Gamma (\beta +\epsilon )\right)}^{2}-(1+3\lambda )\Gamma (2\beta +\epsilon )\Gamma (\epsilon ))\\ -2C_{2}^{\zeta }(\tau ){\left(1+\lambda \right)}^{2}\Gamma (2\beta +\epsilon ){\left(\Gamma (\epsilon )\right)}^{2} \end{array}}+\frac{C_{1}^{\zeta }(\tau )\Gamma (2\beta +\epsilon )e^{2\mu }(c_{2}-d_{2})}{4\mu (1+2\lambda )\Gamma (\epsilon )}\\ & =C_{1}^{\zeta }(\tau )([h(\eta )+\frac{\Gamma (2\beta +\epsilon )e^{2\mu }}{4\mu (1+2\lambda )\Gamma (\epsilon )}]c_{2}+[h(\eta )-\frac{\Gamma (2\beta +\epsilon )e^{2\mu }}{4\mu (1+2\lambda )\Gamma (\epsilon )}]d_{2}), \end{array}$$

where

$$h(\eta )=\frac{(1-\eta ){\left(C_{1}^{\zeta }(\tau )\right)}^{2}e^{2\mu }\Gamma (2\beta +\epsilon ){\left(\Gamma (\beta +\epsilon )\right)}^{2}}{\begin{array}{c} 2{\left(C_{1}^{\zeta }(\tau )\right)}^{2}\Gamma (\epsilon )(2\mu (1+2\lambda ){\left(\Gamma (\beta +\epsilon )\right)}^{2}-(1+3\lambda )\Gamma (2\beta +\epsilon )\Gamma (\epsilon ))-2C_{2}^{\zeta }(\tau ){\left(1+\lambda \right)}^{2}\Gamma (2\beta +\epsilon ){\left(\Gamma (\epsilon )\right)}^{2} \end{array}}.$$

Then, in view of (1.1), and using (3.5), we conclude that

$$\left|a_{3}-\eta a_{2}^{2}\right|\leq \left\{ \begin{array}{cc} \frac{\tau \left|\zeta \right|\Gamma (2\beta +\epsilon )e^{2\mu }}{\mu (1+2\lambda )\Gamma (\epsilon )} & \text{ if}\;0\leq \left|h(\eta )\right|\leq \frac{\Gamma (2\beta +\epsilon )e^{2\mu }}{4\mu (1+2\lambda )\Gamma (\epsilon )}\\ 4\tau \left|\zeta \right|\left|h(\eta )\right| & \text{ if}\;\left|h(\eta )\right|\geq \frac{\Gamma (2\beta +\epsilon )e^{2\mu }}{4\mu (1+2\lambda )\Gamma (\epsilon )}. \end{array}\right.$$

 □

Corollary 3.1 *If* f∈Δ *is expressed by the equation* (2.1) *and belongs to the class* $N_{\Delta }^{0}(\beta ,\epsilon ,\mu ,G_{\zeta }(\tau ,\varphi ))$ *, then,*

$$\left|a_{2}\right|\leq \frac{2\tau |\zeta |e^{\mu }\Gamma (\beta +\epsilon )\sqrt{2\tau |\zeta |\Gamma (2\beta +\epsilon )}}{\sqrt{\begin{array}{c} \left|2\zeta^{2}\tau^{2}\Gamma (\epsilon )(4\mu {\left(\Gamma (\beta +\epsilon )\right)}^{2}-2\Gamma (\epsilon )\Gamma (2\beta +\epsilon ))+\zeta \Gamma (2\beta +\epsilon ){\left(\Gamma (\epsilon )\right)}^{2}(1-2\tau^{2})\right| \end{array}}}$$

$$\left|a_{3}\right|\leq \frac{2\tau |\zeta |e^{\mu }\Gamma (\beta +\epsilon )\sqrt{2\tau |\zeta |\Gamma (2\beta +\epsilon )}}{\sqrt{\begin{array}{c} \left|2\zeta^{2}\tau^{2}\Gamma (\epsilon )(4\mu {\left(\Gamma (\beta +\epsilon )\right)}^{2}-2\Gamma (\epsilon )\Gamma (2\beta +\epsilon ))+\zeta \Gamma (2\beta +\epsilon ){\left(\Gamma (\epsilon )\right)}^{2}(1-2\tau^{2})\right| \end{array}}}+\frac{\tau |\zeta |\Gamma (2\beta +\epsilon )e^{2\mu }}{\mu \Gamma (\epsilon )},$$

*and*

$$\left|a_{3}-\eta a_{2}^{2}\right|\leq \left\{ \begin{array}{cc} \frac{\tau \left|\zeta \right|\Gamma (2\beta +\epsilon )e^{2\mu }}{\mu \Gamma (\epsilon )} & \;\text{if}\;0\leq \left|h_{1}(\eta )\right|\leq \frac{\Gamma (2\beta +\epsilon )e^{2\mu }}{4\mu \Gamma (\epsilon )}\\ 4\tau \left|\zeta \right|\left|h_{1}(\eta )\right| & \;\text{if}\;\left|h_{1}(\eta )\right|\geq \frac{\Gamma (2\beta +\epsilon )e^{2\mu }}{4\mu \Gamma (\epsilon )}, \end{array}\right.$$

*where*

$$h_{1}(\eta )=\frac{2\zeta \tau^{2}(1-\eta )e^{2\mu }\Gamma (2\beta +\epsilon ){\left(\Gamma (\beta +\epsilon )\right)}^{2}}{\begin{array}{c} 2\zeta \tau^{2}\Gamma (\epsilon )(4\mu {\left(\Gamma (\beta +\epsilon )\right)}^{2}-3\Gamma (2\beta +\epsilon )\Gamma (\epsilon ))+\Gamma (2\beta +\epsilon ){\left(\Gamma (\epsilon )\right)}^{2}(1-2\tau^{2}) \end{array}}.$$

Corollary 3.2 *If* f∈Δ *is expressed by the equation* (2.1) *and belongs to the class* $N_{\Delta }^{1}(\beta ,\epsilon ,\mu ,G_{\zeta }(\tau ,\varphi ))$ *, then,*

$$\left|a_{2}\right|\leq \frac{\tau |\zeta |e^{\mu }\Gamma (\beta +\epsilon )\sqrt{2\tau |\zeta |\Gamma (2\beta +\epsilon )}}{\sqrt{\begin{array}{c} \left|\zeta^{2}\tau^{2}\Gamma (\epsilon )(6\mu {\left(\Gamma (\beta +\epsilon )\right)}^{2}-5\Gamma (\epsilon )\Gamma (2\beta +\epsilon ))+\zeta \Gamma (2\beta +\epsilon ){\left(\Gamma (\epsilon )\right)}^{2}(1-2\tau^{2})\right| \end{array}}}$$

$$\left|a_{3}\right|\leq \frac{2\tau |\zeta |e^{\mu }\Gamma (\beta +\epsilon )\sqrt{2\tau |\zeta |\Gamma (2\beta +\epsilon )}}{\sqrt{\begin{array}{c} \left|2\zeta^{2}\tau^{2}\Gamma (\epsilon )(4\mu {\left(\Gamma (\beta +\epsilon )\right)}^{2}-2\Gamma (\epsilon )\Gamma (2\beta +\epsilon ))+\zeta \Gamma (2\beta +\epsilon ){\left(\Gamma (\epsilon )\right)}^{2}(1-2\tau^{2})\right| \end{array}}}+\frac{\tau |\zeta |\Gamma (2\beta +\epsilon )e^{2\mu }}{3\mu \Gamma (\epsilon )},$$

*and*

$$\left|a_{3}-\eta a_{2}^{2}\right|\leq \left\{ \begin{array}{cc} \frac{\tau \left|\zeta \right|\Gamma (2\beta +\epsilon )e^{2\mu }}{3\mu \Gamma (\epsilon )} & \;\text{if}\;0\leq \left|h_{1}(\eta )\right|\leq \frac{\Gamma (2\beta +\epsilon )e^{2\mu }}{12\mu \Gamma (\epsilon )}\\ 4\left|\zeta \right|\left|h_{2}(\eta )\right| & \;\text{if}\;\left|h_{2}(\eta )\right|\geq \frac{\Gamma (2\beta +\epsilon )e^{2\mu }}{12\mu \Gamma (\epsilon )}, \end{array}\right.$$

*where*

$$h_{2}(\eta )=\frac{\zeta \tau^{2}(1-\eta )e^{2\mu }\Gamma (2\beta +\epsilon ){\left(\Gamma (\beta +\epsilon )\right)}^{2}}{\begin{array}{c} 12\zeta \tau^{2}\Gamma (\epsilon )(\mu {\left(\Gamma (\beta +\epsilon )\right)}^{2}-\Gamma (2\beta +\epsilon )\Gamma (\epsilon ))+2\Gamma (2\beta +\epsilon ){\left(\Gamma (\epsilon )\right)}^{2}(1-2\tau^{2}) \end{array}}.$$

## 4 Conclusion

In our study, we presented a novel subclass within the domain of normalized analytic bi-univalent functions. This distinct subclass integrates Gegenbauer polynomials with the Mittag-Leffler-type Borel distribution, presenting a unique approach to function analysis. We established upper bounds for the Taylor-Maclaurin coefficients $\left|a_{2}\right|$ and $\left|a_{3}\right|$, followed by tackling the Fekete-Szegö inequality problem. Additionally, by selecting suitable parameter values, we obtained several new results.

## CRediT authorship contribution statement

**Abdulmtalb Hussen:** Writing – review & editing, Writing – original draft, Visualization, Validation, Resources, Project administration, Investigation.

## Declaration of Competing Interest

The authors declare the following financial interests/personal relationships which may be considered as potential competing interests: Abdulmtalb Hussen reports a relationship with Navajo Technical University that includes.

## Data Availability

Data sharing is not relevant to this article as no datasets were generated or analyzed during the course of this study.
